# Adaptation and psychometric evaluation of the Natural Disasters Picture System (NDPS) to the Spanish population

**DOI:** 10.3389/fpsyg.2026.1911152

**Published:** 2026-09-16

**Authors:** Danae A. Mendoza, José M. Reales, Soledad Ballesteros

**Affiliations:** 1 Doctoral School, Universidad Nacional de Educación a Distancia (UNED), Madrid, Spain; 2 Department of Methodology of Behavioral Sciences, Facultad de Psicología, Universidad Nacional a Distancia (UNED), Madrid, Spain; 3 Department of Basic Psychology II, Facultad de Psicología, Universidad Nacional de Educación a Distancia (UNED), Madrid, Spain

**Keywords:** emotions, natural disasters, picture database, SAM, volcanoes, NDPS

## Abstract

**Introduction:**

Climate change is causing an increase in natural disasters and extreme weather events. This threat is impacting the mental health of those who experience it. However, empirical research in the Spanish context is limited by the lack of culturally adapted tools.

**Methods:**

In this study, we have adapted and validated with a Spanish sample composed by 73 participants the Natural Disasters Picture System (NDPS). The methodology incorporated Exploratory Factor Analysis (EFA), Mixed Linear Models (MLM) with intercepts for subjects and stimuli, and predictive classification using Machine Learning (Random Forest).

**Results:**

The results demonstrated the cross-cultural robustness of the original four clusters (“Frightening,” “Desolation,” “Neutral” and “Pleasant/Aesthetic”). Additionally, we identified a unidimensional latent factor and a significant perceptual overlap between “Frightening” (fear) and “Desolation” (sadness) in the Machine Learning Model.

**Discussion:**

The NDPS provides a set of visual stimuli that can be used to investigate risk perception and the elicitation of emotions. Despite the appropriate distribution of commonalities and the accuracy of MLMs, that support the instrument's properties, we acknowledge the possible limitation sample.

## Introduction

The increasing frequency of natural disaster linked to climate change highlights the need to gain a deeper understanding of how this affects perceived risk. Natural disaster are events of extreme impact on the lives of people and communities that experience them, and trigger negative emotional responses, psychological distress and acute stress, that can evolve into post-traumatic stress disorder (Mendoza et al., 2024).

The perception of risk in the face of natural disasters is fundamentally shaped by rapid emotional reactions. The processing of threatening stimuli is guided by the “affective heuristic” (experiential system), which is defined by Slovic et al. (2004) as the use of the general feeling-“good” or “bad”- as a mental shortcut or cue for assessing danger. The effectiveness of the affective heuristic depends on the qualities of real or imagined stimuli in evoking affective images that have both perceptual (sounds, smells…) and symbolic (words, ideas…) interpretations. These mental representations may consist of affective dimensions-how we *feel* about the risk- and instrumental dimensions-how *assess* the risk through cost-benefit analysis—. Specifically, when an action or object is perceived as “good,” people tend to judge its benefits as high and its risk as low; conversely, if it is perceived as “bad,” the benefits are judged as low and the risks as high.

In the context of natural disasters, the literature identified various emotions that appear to influence risk assessment. In Slovic (1987) the perception of risk is affected by the affective dimension of “dread” characterized by a lack of control, catastrophe and the inevitability of the consequences. According to Böhm (2003), emotional reactions to environmental risk are multidimensional in nature. In this study, an assessment based on the dimension of “consequence” and “ethics” revealed a distinct pattern for each environmental risk in terms of emotional response and cognitive judgment. For example, chemical spills elicit the most intense emotions related to ethics, whilst this type of emotion is less intense for natural hazards such as volcanic eruptions. Another study suggest that affect (feelings) accounts for a significant proportion for the variance in risk perception, even when cognitive and sociodemographic factors are controlled for (van der Linden, 2015). In summary, affect acts as a central determinant in the formation of risk perception.

Emotions are a complex psychological phenomenon, and the literature contains numerous definitions and theoretical approaches. Standardized Emotion Elicitation Databases (SEEDs) enable emotions to be studied systematically by replicating real-life emotions in a controlled environment. Branco et al. (2023) noted in their review that the validation process tends to involve a combination of self-report and physiological data, or the collection of self-report data only. Self-report data may use a Likert-type scale to measure affective dimensions and/or involve categorizing stimuli based on the emotions they evoke. Moreover, physiological data may include Skin Conductance Level (SCL), Heart Rate Variability (HRV) and Electroencephalography (EEG).

The most widely used SEEDs are the International Affective Picture System [IAPS; Lang et al. (2008)], the Nencki Affective Picture System [NAPS; Marchewka et al. (2014)] and the Geneva Affective Picture Database [GAPED; Dan-Glauser and Scherer (2011)]. Several authors have documented (Coan and Allen, 2007) the usefulness of these picture databases in fields as diverse as affective neuroscience, the assessment of attentional biases, clinical research, psychophysiology reactivity and the analysis of risk perception. Unfortunately, the pictures in these databases lack a comprehensive and ecologically valid category for natural catastrophes. Therefore, applicability to the specific field of natural disasters investigation is limited due to insufficient thematic images. This lack is especially important when searching for pictures of volcanic phenomena, whose diversity of manifestations-lava flows, lahars, ash falls, etc.,-requires a broader repository than the one offered by most of the generalist picture databases. To fill this gap, Merlhiot et al. (2018) developed the Natural Disaster Picture System (NDPS), which includes representations of natural disasters and environmental degradation selected to induce different emotional responses.

An important issue concerning emotions, that remains the subject of lively debate amongst academics, is the universal nature of emotions vs. their cultural specificity. Although there is a universal biological framework for the facial recognition of basic emotions (Russell, 1994), the perception of risk and the emotional response are constructs that acquire meaning within a specific cultural context. According to Scherer et al.'s (2001) Component Process Model (CPM), emotion are the result of a series of appraisals, including the individual analyses, the relevance of the event, their capacity to cope with it, and its moral or social significance. In other words, the emotions evoked by a visual stimulus do not depend entirely on its visual qualities, but on the meaning attributed to it by the individual and the community. In line with the guidelines set out by van de Vijver and Tanzer (2003) that defines and addresses primary biases -construct, method, and item- and levels of equivalence -structural, measurement unit, and full scale- in cross-cultural assessment, the evaluation of the NDPS in Spain is necessary to ensure the ecological validity and psychometric equivalence of the visual stimuli that constitute the picture database when evoking a specific emotional response across cultures.

The main objective of this study is to conduct the cross-cultural adaptation and psychometric evaluation of the NDPS for its use in the Spanish population. The specific objectives of the present study are: (1) to establish affective norms for the classic dimensions of Valence, Arousal and Dominance, and the new dimension of Certainty for each visual stimuli; (2) to verify the underlying structure of the pictures through the analysis and exploration of the clusters and classes proposed by Merlhiot et al. (2018) in the original study; and (3) to assess the psychometric equivalence of the visual stimuli in the Spanish context.

## Methods

The picture database used, originally called the *Natural Disasters Picture System* (NDPS), was translated into Spanish as “Sistema de Imágenes de Desastres Naturales (SIDN),” a name that is recommended for citation in Spanish contexts.

### Description of the original image database

The picture database used in this study is comprises 154 pictures depicting various types of natural disasters (e.g., earthquakes, hurricanes, tornadoes), with a particularly large section dedicated to volcanic hazards (explosions, lava flows, ash and lapilli falls, and pyroclastic flows). The set was divided into two subsets, one of 72 pictures (Group A) and the other of 83 pictures (Group B).

The pictures come from open and specialized sources-Flickr, Wikimedia Commons, USGS (United States Geological Survey), USDA (United States Department of Agriculture)-and from personal archives of volcanologists and photographers. All the pictures were in the public domain, under a Creative Commons license, or with the permission of the authors for its use in research. Pictures 19 was removed due to an error in copyright attribution in the original version.

To ensure homogeneity and visual quality, all images were processed in the original version with RawTherapee [version 4.0.11.8; (The RawTherapee, n.d.)], correcting sharpness, exposure (highlights, shadow, contrast), white balance, resolution and framing. A minimum resolution of 1,200 × 800 pixels was established. The detailed categorization of each picture (identifier, cluster, classes, and other specific characteristics) is listed in Appendix 1.

### Adaptation process to the Spanish population

#### Evaluation instrument

The instrument selected for participants' responses was the Self-Assessment Manikin (SAM) scale (Bradley and Lang, 1994; Morris, 1995), using as a reference the Spanish and computerized version adapted by Redondo et al. (2005). SAM assesses three basic emotional dimensions in line with valuation theories (Scherer et al., 2001; Ellsworth and Scherer, 2002). These dimensions are Valence (pleasant-unpleasant), Arousal (calm-excited), and Dominance (dominated-dominant).

Each dimension was presented on a 9-point scale with 5 mannequin-like figures and spaces in between. In the original version, a new dimension, “Certainty,” was introduced because the perception of certainty according to the Component Process Model (CPM) provides a key component for more effectively categorizing discrete emotions (Scherer et al., 2001) induced by each picture of the NDPS, and because its evaluation would benefit studies on judgements, decision-making and communications theories (Tiedens and Linton, 2001; Loewenstein et al., 2001; Kramer, 2015). This new dimension was designed with a photo editor [GIMP, version 2.8.6; (GIMP Team, n.d.)], maintaining the visual style of the SAM. In this adaptation, “Certainty” was conserved, with the following assessment: Extreme “Uncertain” or feeling of not understanding the situation, not foreseeing the outcome or not anticipating the future and the extreme “Certainty” as feeling of understanding the situation, foreseeing the outcome and anticipating the future.

#### Translation and linguistic adaptation process

To ensure the semantic and cultural equivalence of the scale, the descriptive texts associated with the dimensions were reviewed and translated into Spanish. We prioritized the names established in the validated Spanish adaptation of the SAM, and for the descriptive texts, we attempted to follow the same tone and vocabulary, ensuring terminological consistency with previous studies. For the Certainty dimension, as there was no previous version in Spanish, we conducted a direct translation from the original English text by bilingual authors (DM and JMR). Subsequently, we conducted a consensus synthesis, followed by an independent back-translation to verify conceptual equivalence. The final text was reviewed by DM, JMR, and SB, who evaluated the clarity, accuracy and cultural appropriateness of the descriptors before their final inclusion in the assessment task. Once the final Spanish version had reached consensus, we conducted preliminary tests on the most common devices used by participants to access the task, such as PCs, laptops and smartphones (Android and IOS), to ensure that the descriptive texts are displayed correctly and are easy to understand.

#### Experimental procedure

The adaptation followed the standardized instructions of the original protocol, similar to those used in the validation of the IAPS (Lang et al., 2008). In this study, we replaced the instructor with explanatory screens in order to achieve a wider and more representative sample of the Spanish populations. Each dimension was presented with its name -heading-, representative labels, SAM figures, and a numerical scale (from 1 to 9).

The task sequence was as follows: first, a preparation message appeared on the screen for 5 s (“Prepare to evaluate the next image”) followed by the picture presentation that lasted 6 seconds. When the 6 s elapsed, the participant had to conduct sequentially the dimensional evaluation of each of the four scales described above for 20 s (5 s per dimension).

If the allotted time for response was exceeded, an alert message appeared on the computer screen: “Please respond quickly” (elapsed 2.5 s, the screen moved on to the assessment of the next dimension). The task began with a training phase with 6 neutral pictures and between the third and fourth trials of this training phase, the order of the dimensions was changed.

The duration of the task was approximately 30 min. To prevents the effects of fatigue, rumination or presentation order biases, a multilevel randomization and counterbalancing design was incorporated. To achieve this, the set of pictures was randomized. Each picture set was divided into 4 subsets (~20 pictures) using RStudio (R Studio Team, n.d.) with the “deck” command. Then, each subset was randomized again in PsychoPy (2019) and generating a unique sequence per session and participant. The request to assess dimensions in each trial was rotated into four distinct sequences. These sequences were: (1) Valence-Arousal-Dominance-Certainty; (2) Arousal-Valence-Certainty-Dominance; (3) Valence-Certainty-Dominance-Arousal; and (4) Dominance-Certainty-Valence-Arousal, which changed every 18 pictures in Group A and every 21 in Group B.

The task was programmed in PsychoPy [version 2024.2.4; (Peirce et al., 2019)] and hosted in Pavlovia (Pavlovia, n.d.), an online platform for conduction of studies developed with PsychoPy. A desktop or laptop computer with mouse was recommended for its completion. The sequence of the assessment task is illustrated in Appendix 1.

#### Participants recruitment, inclusion criteria, and sample characteristics

##### Recruitment

Participants were recruited online between December 2024 and August 2025, using two main channels, the diffusion on professional social networks of the first author (DM), specifically through LinkedIn profile, with posts tagged with hashtags referring to natural disasters, and sending direct invitations to personal and professional contacts who met the inclusion criteria, requesting their voluntary collaboration.

Access to the experimental task was provided via a link to the Pavlovia platform, accompanied by a brief description of the study, its objectives, and the conditions of participation in an advertisement format. Before starting the experimental task, we ensured that each participant received explanatory screens detailing the following aspects: the nature and objectives of the study, the kind of pictures to assess, warning of their potentially disturbing nature, the voluntary nature of participation, the possibility of leaving the study at any time, and the guarantees of confidentiality and anonymity in the data processing.

Consent was formalized by clicking the “I agree to participate” button, which was a prerequisite for accessing the experimental task. Figure 1 displays the Flow Diagram including recruitment, group allocation and analyzed sample.

**Figure 1:**
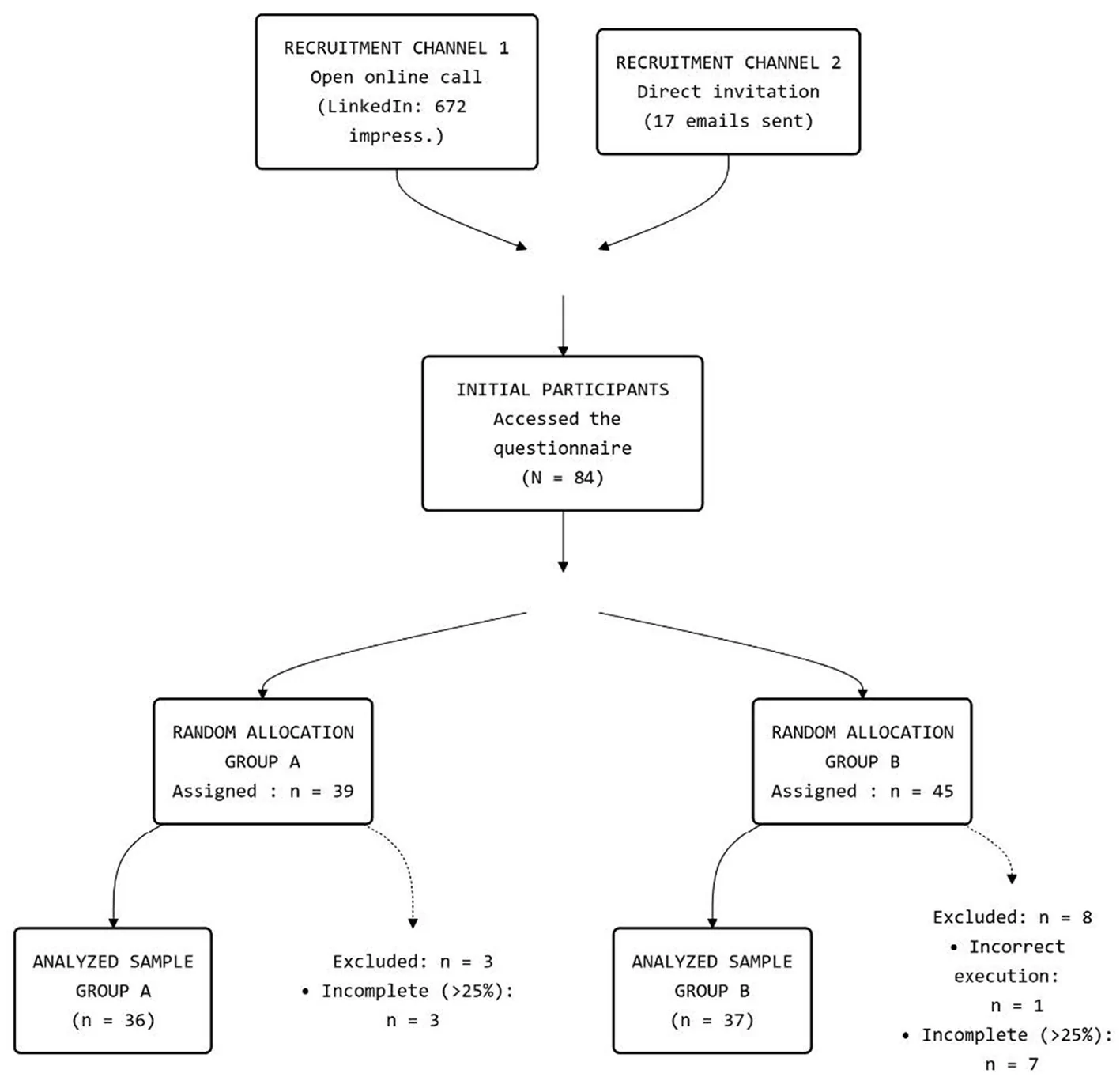
Flow diagram of participant recruitment, group allocation, and final analysis.

##### Inclusion and exclusion criteria

The inclusion criteria were to be 18 years of age or older at the time of participation, Spanish or legal residence in Spain, and to provide informed consent after receiving detailed information about the nature of the study and the kind of pictures that would be presented. Participants who completed at least 75% of the rating for the assigned picture set were included in the study.

Participants who submitted identical responses in all assessments were excluded from the final analysis, as it was considered that they were unable to perform the task correctly (*n* = 1). Likewise, and to ensure the reliability of the response profiles, those who left more than 25% of the assessments uncompleted (*n*=11) were excluded from the final analysis. Finally, as a novel element in this adaptation and given its relevance for the interpretation of emotional and cognitive responses, a question was added to record whether the participant had personally experienced a natural disaster.

##### Characteristic of the sample

The final sample consisted of 73 participants (53 females, 17 males, 2 non-binary people, and 1 who preferred not to disclose its gender) with a mean age of 37.2 years (*SD* = 11.3). The distribution by groups was as follows:

- Group A (72 pictures, *n* = 36): 75% female, 22.22% male and 2.78% non-binary; mean age = 34.6 years (SD = 8.20); 3 participants with direct experience in natural disasters.
- Group B (83 pictures, *n* = 37): 70.27% female, 24.32% male, 2.70% non-binary and 2.70% preferred not to say its gender; mean age = 39.7 years (*SD* = 13.3); 5 participants with direct experience in natural disasters.

This sample size was considered methodologically appropriate because it is consistent with, or even higher than, the standards of relevant international reference picture databases, such as the GAPED normative study [*N* = 60; (Dan-Glauser and Scherer, 2011)]. To ensure equivalence between the subsamples a homogeneity analysis was conducted comparing age and gender distribution. The results indicate that there is no statistically significant difference in gender (*p* = 0.786), but there is a marginally significant difference in terms of age (*p* = 0.053), suggesting that the division of the sample did not introduce any confounding variables into the subsequent psychometric analysis. To consult the homogeneity matrix table, see the Appendix 1.

##### Statistical analysis

All statistical analyses were performed with RStudio software [version 2025.05.1; (R Studio Team, n.d.)]. After initial data processing with the **tidyverse** package, descriptive statistics and Pearson correlations were calculated for the scores of the four dimensions on each picture. The effect of the group variable (Group A or B) on the participants' emotional response was assessed with Inter-subject ANOVA. Inter-rater reliability was determined by the Intraclass Correlation Coefficient (ICC_2, k_). To explore latent cluster based on the emotional responses (without imposing predefined clusters), we performed Hierarchical Cluster Analysis (HCA) to determine the optimal number of clusters, followed K-means to optimize the assignment of pictures to clusters empirically. Principal Component Analysis (PCA) and Exploratory Factor Analysis (EFA) were applied to define the metrical structure. PCA reduces the total variance in the data and confirms the appropriate metric. EFA was used to model the latent variables to identify the underlying theoretical structure of the affective dimensions. The evaluation of the emotional structure of NDPS was carried out using Mixed Linear Models (MLM) using the **lm4** package with *post-hoc* comparisons (**emmeans** package) applying Tukey's adjustment and the **effectsize** package for estimating effect size. MLM controls individual variability among participants and visual stimuli by including them as random effects. This facilitates the estimation of the true differences when comparing scores across the various cluster and classes of pictures. Additionally, we conducted complementary analyses to verify the robustness to the stimulus classification and to overcome the limitations of classical psychometrics. The aim of the inclusion of Machine Learning classification model (Random Forest) with the **caret** package was understand whether participants' scores on the affective dimensions have sufficient capacity to automatically discriminate, predict and classify pictures into their respective clusters. Finally, to explore and visualized the natural form of the stimuli, we performed the Uniform Manifold Approximation and Projection (UMAP) algorithm and t-Distributed Stochastic Neighbor Embedding (t-SNE), implemented in the **uwot** package. These non-linear dimensionality reduction techniques mapped and visualized the spatial structure and clustering of the pictures in a two-dimensional space.

## Results

The analysis of the NDPS evaluation results was structured in several phases. First, we examined the psychometric properties of the instrument. Second, we explored the structure of the picture stimuli in the Spanish sample. Third, the evaluation of the original emotional classification using advanced statistical models was conducted. Finally, we conducted complementary classification and visualization analyses to better understand the database.

### Preliminary and reliability analyses

The descriptive analyses of the pictures and clusters are available in Appendix 2. In addition, the relationships between the four dimensions (Valence, Arousal, Dominance and Certainty) and the reliability of the measurements were examined.

### Analysis of structural similarity of the groups

We checked whether the emotional dimensions maintained a similar structural relationship in both groups of stimuli (A and B). We used the mean scores aggregated per stimulus (pictures), obtained by averaging responses of all participants for each individual picture. The correlations coefficients were extremely high in both matrices (see Table 1), and consistent in the strength and direction of the relationship, with differences considered minimal in term of factorial structure. This justifies combining the data for the PCA and EFA analyses.

**Table 1:** Pearson correlation matrix between dimensions (Group A *vs*. Group B).

Dimensions	1	2	3	4
1. Valence	-	−0.990^***^	0.982^***^	0.983^***^
2. Arousal	−0.958^***^	-	−0.988^***^	−0.987^***^
3. Dominance	0.900^***^	−0.944^***^	-	−0.990^***^
4. Certainty	0.924^***^	−0.949^***^	0.953^***^	-

Values below the diagonal correspond to Group A (*n* = 36); values above the diagonal correspond to Group B (*n* = 37). ^***^ *p* < 0.001.

Subsequently, we performed an inter-subject ANOVA using the mean scores per participant, calculated as the average of an individual's response to all stimuli in their corresponding set. This revealed that groups A and B were equivalent in Arousal (*p* = 0.698), Dominance (*p* = 0.465), and Certainty (*p* = 0.126). However, a statistically significant effect was found in the Valence dimension [*F*(1, 71) = 6.612, *p* = 0.0122, η^2^ = 0.0852]. This difference is most likely caused by an imbalance in the selection of the emotional content of the stimuli that make up each group, rather by an inherent difference between the participants. For more information, see Table 2, and for the boxplot, see Appendix 3.

**Table 2:** Inter-subject ANOVA comparing dimension by Group (Group A *vs*. Group B).

Dimensions	*F*	**d.o.f**.	***p***	*η^2^*
Valence	6.612	71	0.0122^*^	0.0852
Arousal	0.151	71	0.698	0.0021
Dominance	0.540	71	0.465	0.0076
Certainty	2.394	71	0.126	0.0326

*F*, ratio between the variance attributable to the difference among the groups and the residual variance. d.o.f, degrees of freedom. η^2^, effect size index. ^*^p < 0.05.

Based on previous results, and verifying that there is stability between groups, we decided that it is appropriate to combine both groups with the aim to calculate the global norms for the NDPS. Thus, from now on, the results are referring to the complete set of pictures.

### Correlation analysis between dimensions

We calculated the Pearson's correlation matrix between the four evaluated dimensions (Valence, Arousal, Dominance and Certainty) using the aggregate mean scores for each stimulus. Table 3 presents the results that revealed theoretically coherent patterns. We found a negative correlation between Valence and Arousal (*r* = −0.77), indicating that the more unpleasant pictures generated greater physiological activation. In turn, Valence showed positive correlations with Dominance (*r* = 0.73) and Certainty (*r* = 0.75). Arousal correlated negatively with Dominance (*r* = −0.75) and Certainty (*r* = −0.74), suggesting that the most activating situations were associated with a lower perception of certainty and control.

**Table 3:** Pearson correlation matrix, means and standard deviations for the dimensions.

Dimension	M	SD	1	2	3	4
1. Valence	3.74	1.73	-			
2. Arousal	5.86	1.78	−0.77^***^	-		
3. Dominance	4.21	1.83	0.73^***^	−0.75^***^	-	
4. Certainty	4.09	1.78	0.75^***^	−0.74^***^	0.79^***^	-

*M*, Means, SD, Standard Deviation. *N* = 5.627. ^***^ *p* < 0.001.

### Inter-rater reliability analysis (ICC)

We evaluated the consistency of the rating among the 73 participants using the Intraclass Correlation Coefficient (ICC_2, k_) using each participant's raw scores for each stimulus in the four dimensions. The results showed moderate reliability for Valence (ICC_2, k_ = 0.53) and Arousal (ICC_2, k_ = 0.48), and lower reliability for Dominance (ICC_2, k_ = 0.42) and Certainty (ICC_2, k_ = 0.43) at the individual level. However, the mean reliability was excellent for all dimensions (ICC_2, k_ > 0.98). This indicates that the aggregated mean scores per picture, used in subsequent analyses, are extremely stable and reliable.

### Analysis of the stimuli structure

Below, we describe the analysis of the stimulus structure, highlighting the most relevant results. In all multivariate analyses (HCA, K-Means, PCA and EFA), the unit of analysis was the stimulus (156 pictures), using its means scores obtained from the total sample of participants (*N* = 73). Individual subject scores were not used to avoid statistical noise derived from inter-personal variability. For an expanded version of the stimulus structure revealed by the previous analysis, see Appendix 4.

### Exploratory cluster analysis

We also explored the natural clustering of stimuli in the Spanish sample. The elbow and silhouette methods suggested that the most parsimonious and statistically optimal solution was two clusters (*k* = 2), indicating a primary distinction between high and low-emotional impact imaging.

However, Hierarchical Cluster Analysis (HCA) revealed a more nuanced structure. As shown, the dendrogram in the hierarchical structure allows for a coherent division into finer clusters (see Figure 2). The evaluation of the Within-Cluster Sum Squares (WSS) indicated that *K* = 4 provided a significantly more robust “elbow,” yielded a further 38% in variance (WSS = 59.11). A more intensive exploration using *K*-means can be found in Appendix 4.

**Figure 2:**
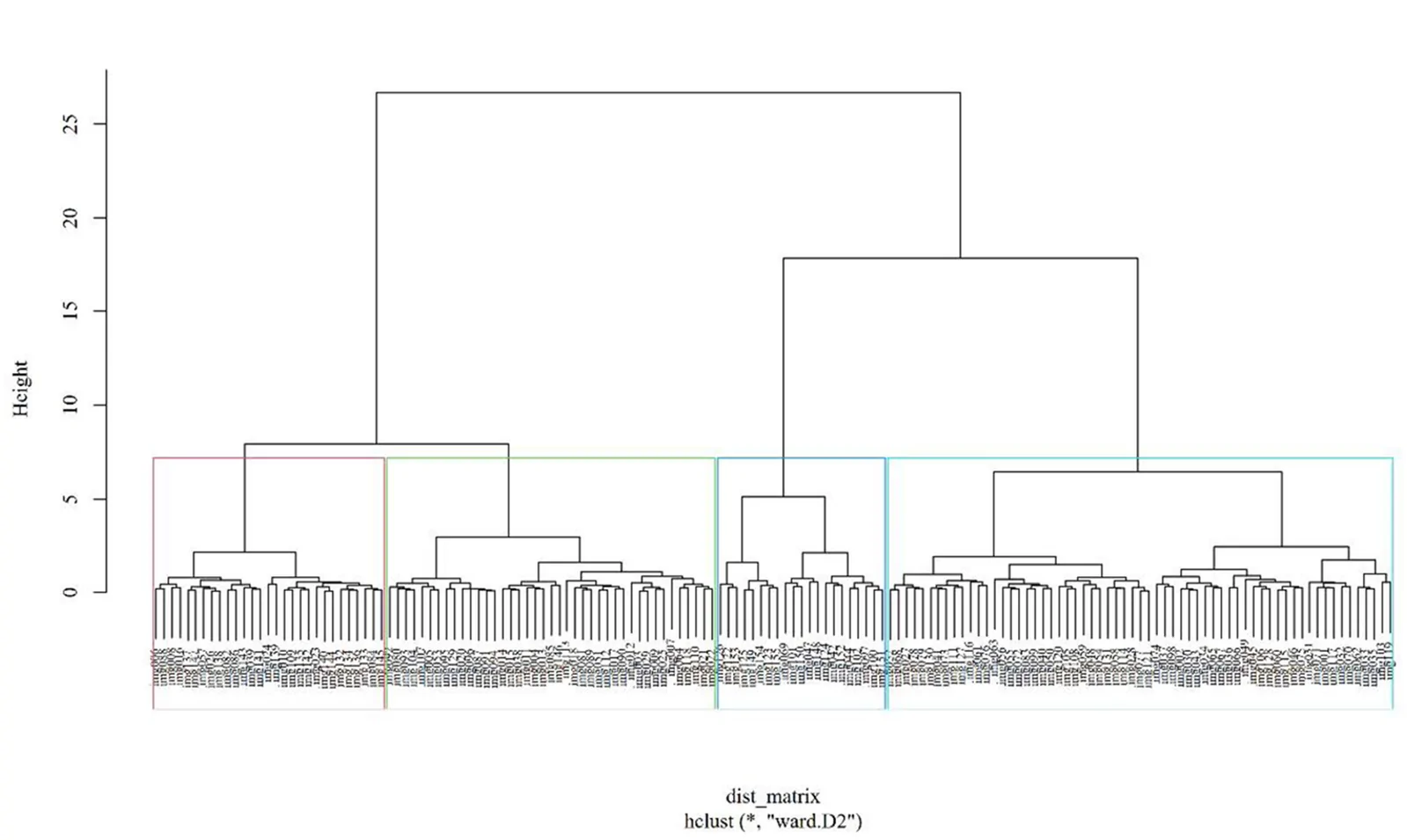
Dendrogram of Hierarchical Cluster Analysis (HCA).

Therefore, as can be seen in the dendrogram and cluster analysis, and with the intention of maintaining theoretical equivalence with the original version of Merlhiot et al. (2018) composed of the Desolation, Neutral, Frightening, and Pleasant/Aesthetic clusters, a four-cluster solution was selected as the optimal structure for the subsequent Mixed Linear Models (MLMs) analysis.

### Analysis of the dimensional structure

Principal Component Analysis (PCA) indicated a strong one-dimensionality in the data. The first principal component explained 97.86% of the total variance. Prior to factor analysis, the suitability of the data matrix for a PCA was assessed using Barlett's sphericity test and the Kaises-Meyer-Olkin test. The results supported the adequacy of the data for factor analyses with an overall value of KMO = 0.84 and a statistically significant Barlett's test (χ^2^ = 1449.21, *df* = 6, *p* < 0.001). The Exploratory Factor Analysis (EFA) confirmed that a single factor accounted for almost all the variance, with all the dimensions showing high factor loadings (loads > 0.98) and communalities greater than 0.96. Following MacCallum et al. (1996), being the mean communality of $h^{2}=0.972$, the sample sizes should be about 35 participants, well below our sample size. The internal consistency as measured by Cronbach's Alpha (α = 0.992) reinforced this unidimensional structure. These results suggest that, although four distinct emotional profiles can be identified in the answers to the pictures, there is a high degree of convergence on a general factor. However, the high correlation between the dimensions does not necessarily imply that the existence of underlying psychological constructs with theoretical validity.

According to the factor scores, the picture 122 showed the highest level of overall affective impact (Factor Score = 2.77), while the picture 142 recorded the lowest score for emotional reactivity (Factor Score = −1.468). To facilitate the selection of stimuli in future research, Appendix 5 sets out the factor scores derived from the AFE for each picture.

### Evaluation of emotional profiles

We used Mixed Linear Models (MLMs) to validate the structure of the four clusters from the original study, controlling for nested variability in the data. MLMs allow a more accurate partitioning of variance by including “Participant” and “Stimulus” as crossed random effects. The null models revealed that, for all dimensions, the differences between the pictures explained a substantially higher percentage of the variance than the differences between the participants, with the following results: in Valence, the 53.03% of the variance was due to the pictures, and only 5.36% to the participants. For Arousal, the 48.03% of the variance was due to the pictures, and 8.23% to the participants. For Dominance, the 42.41% was due to the pictures, and the 16.63% to the participants. Finally, for Certainty the 42.71% was due to the pictures, and the 13.91% to the participants. This consistent pattern across all four dimensions confirms that emotional responses were primarily driven by the content of the stimuli, supporting the validity of the instrument.

The introduction of the “Cluster” as fixed-effects predictor in the MLM demonstrated that grouping into 4 clusters is highly effective in discriminating the emotional profiles of the pictures in the Spanish sample (see Table 4). Analysis of variance (using Type III sum of squares) results showed that the main effects of the clusters are highly significant in all dimensions, with values of: Valence [$F\left(3,\;151.87\right)=75.35,p<0.001,\eta_{p}^{2}=0.60$], Arousal [$F\left(3,\;152.96\right)=68.96,p<0.001,\eta_{p}^{2}=0.57$], Dominance [$F\left(3,\;152.33\right)=70.47,p<0.001,\eta_{p}^{2}=0.58$], and Certainty [$F\left(3,\;152.10\right)=70.81,p<0.001,\eta_{p}^{2}=\;0.58$].

**Table 4:** Marginal effects of each dimension per cluster derived from MLMs.

Cluster	Valence	Arousal	Dominance	Certainty	Emotions
Desolation (A)	4.35 (0.15)	5.19 (0.16)	4.91 (0.17)	4.68 (0.16)	Sadness
Neutral (B)	5.95 (0.36)	3.76 (0.36)	6.21 (0.36)	6.00 (0.34)	Neutral/Interest
Frightening (C)	2.97 (0.11)	6.62 (012)	3.46 (0.14)	3.39 (013)	Fear
Pleasant/aesthetic (D)	7.15 (0.34)	2.73 (0.34)	7.12 (0.34)	7.11 (0.32)	Aesthetic emotion

The values represent the Estimated Marginal Mean (EMM), and the values in parentheses correspond to the Standard Error of the mean (SE).

The analysis of the fixed coefficients allowed to identify the “Frightening” cluster as having the greatest negative impact with significantly low scores for Valence (Estimated Marginal Mean, EMM = 2.97), Dominance (EMM = 3.46) and Certainty (EMM = 3.39), and high in Arousal (EMM = 6.62), compared to the other clusters, indicating the generation of fear.

The results of the Turkey-adjusted pairwise revealed that 5 of the 6 pairwise comparison between clusters were statistically significant across the four affective dimensions (*p* < 0.01) and in the theoretically expected direction. The only comparison that did not reach significance in any dimension was that between the Neutral and Pleasant/Aesthetic clusters (Valence = 0.067; Arousal = 0.155; Dominance = 0.207; Certainty = 0.069), indicating that non-negative clusters exhibit a similar reactive profile.

After verifying that the results obtained from the cluster analysis of the Spanish sample are consistent with those from the French sample, we decided to run the MLMs on the nine conceptual classes proposed by Merlhiot et al. (2018), which categorize the pictures according the type of visual content (e.g., presence of victims, destruction of property or volcanic eruptions). The “Class” variable was introduced as fixed-effects.

Analysis of variance (using Type III sums of squares) revealed that Class had a significant main effect on all dimensions evaluated with *p* < 0.001. Specifically: Valence [$F\left(8,\;145.72\right)=64.98,p<0.001,\eta_{p}^{2}=0.78$]; Arousal [$F\left(8,\;146.49\right)=60.03,p<0.001,\eta_{p}^{2}=0.77$]; [$F\left(8,\;145.68\right)=55.90,p<0.001,\eta_{p}^{2}=0.75$]; and Certainty [$F\left(8,\;145.55\right)=58.35,p<0.001,\eta_{p}^{2}=\;0.76$].

As shown in Table 5, the data follow the directionality of the effects in the original version. Specifically, the Victims (VI) class achieved the lowest Valence (EMM = 1.73), and the highest Arousal, closely followed with the Highly Shocking (V) class (EMM_Valence_ = 2.33; EMM_Arousal_ = 6.79). While Pleasant/aesthetic (IX) pictures showed the opposite profile with high level in Valence (EMM = 7.12) and low in Arousal (EMM = 2.15). In the Spanish sample, we find a clear distinction between Desolation/threat (I) and Desolation/loss (II). Desolation/loss (II) showed significantly lower Valence (EMM = 3.91) and high Arousal (EMM = 4.97) compared to Desolation/threat (EMM_Valence_ = 5.29; EMM_Arousal_ = 3.78). However, in the original study, both classes have a more similar scoring profile.

**Table 5:** Marginal effects of each dimension per class derived from MLMs.

Cluster	Class	Valence	Arousal	Dominance	Certainty	Emotions
Desolation (A)	Desolation/threat (I)	5.29 (0.23)	3.78 (0.23)	5.41 (0.23)	4.89 (0.21)	Sadness
	Desolation/loss (II)	3.91 (0.18)	4.97 (0.18)	4.31 (0.18)	3.64 (0.17)	Sadness
Neutral (B)	Neutral (III)	5.93 (0.29)	3.17 (0.30)	5.94 (0.29)	5.34 (0.28)	Neutral/Interest
Frightening (C)	Shocking (IV)	3.13 (0.20)	5.84 (0.20)	3.36 (0.20)	2.82 (0.19)	Fear
	Highly shocking (V)	2.33 (0.30)	6.79 (0.30)	2.45 (0.30)	2.13 (0.28)	Fear
	Victims (VI)	1.73 (0.27)	7.39 (0.27)	1.96 (0.27)	1.62 (0.26)	Fear
	Aftermath/rubble (VII)	2.65 (0.27)	6.29 (0.27)	2.95 (0.27)	2.50 (0.26)	Fear
	Volcanic eruption (VIII)	3.96 (0.20)	5.01 (0.20)	4.12 (0.20)	3.66 (0.19)	Fear
Pleasant/aesthetic (D)	Pleasant/aesthetic (IX)	7.12 (0.28)	2.15 (0.29)	6.85 (0.28)	6.45 (0.27)	Aesthetic emotion

Desolation (**A**) contain 2 classes (Desolation/threat and Desolation/loss); Neutral (**B**) contain 1 class (Neutral); Frightening (**C**) contain 5 classes (Shocking, Highly Shocking, Victims, Aftermath/rubble and Volcanic eruption); and Pleasant/aesthetic (**D**) contain 1 class (Pleasant/aesthetic). The values represent the Estimated Marginal Mean (EMM), and the values in parentheses correspond to the Standard Error of the mean (SE).

Turkey-adjusted pairwise comparisons confirmed significant differences in most classes. The Pleasant/aesthetic (IX) class showed was significantly positive than the Neutral (III) class (*estimate* = 1.10, *p* = 0.031), confirming the generation of a genuinely positive emotional state.

### Exploratory data analysis

Finally, we performed classification and visualization analysis to further explore the feasibility of separating the clusters based on the French version using several algorithms from the Machine Learning (ML) literature. We chose to use clustering to ensure statistical robustness and avoid bias derived from the small size of certain classes. Non-linear dimensionality reduction (UMAP and T-distributed Stochastic Neighbor Embedding (t-SNE) and supervised learning technique (ML) were applied using stimuli as the primary unit of analysis. For more detailed information on exploratory data analyses as a t-SNE graph, consider consulting Appendix 6.

### Classification with machine learning

We trained a Random Forest model to predict the affiliation of a picture in one of the four clusters based on its mean scores for the dimensions. The model achieved an overall accuracy of 75.7% on the test dataset, with a Kappa coefficient of 0.54, indicating moderate performance and a significantly higher level than a random classification. The confusion matrix revealed that the model identified the “Frightening,” “Pleasant/Aesthetic” and “Neutral” clusters with high sensitivity (>91%) but showed difficulties with the “Desolation” cluster (36% sensitivity), which it tended to confuse with “Frightening.” The analysis of the Variable Importance of the Random Forest indicated that Dominance was best predictor in the classification of the pictures into cluster, followed by Arousal and Certainty.

### Visualization with UMAP

The visualization of the data using the UMAP algorithm provided a clear spatial representation of the emotional structure. As illustrated in Figure 3, there is a clear separation between low-impact (“Pleasant/Aesthetic” and “Neutral”) clusters, which form well-defined groupings, and high-impact clusters (“Frightening” and “Desolation”) which showed considerable overlap, and visually corroborates the confusion observed in the Machine Learning model. The t-SNE visualization offered a similar pattern, although with a less defined separation between the cluster (see Appendix 6).

**Figure 3:**
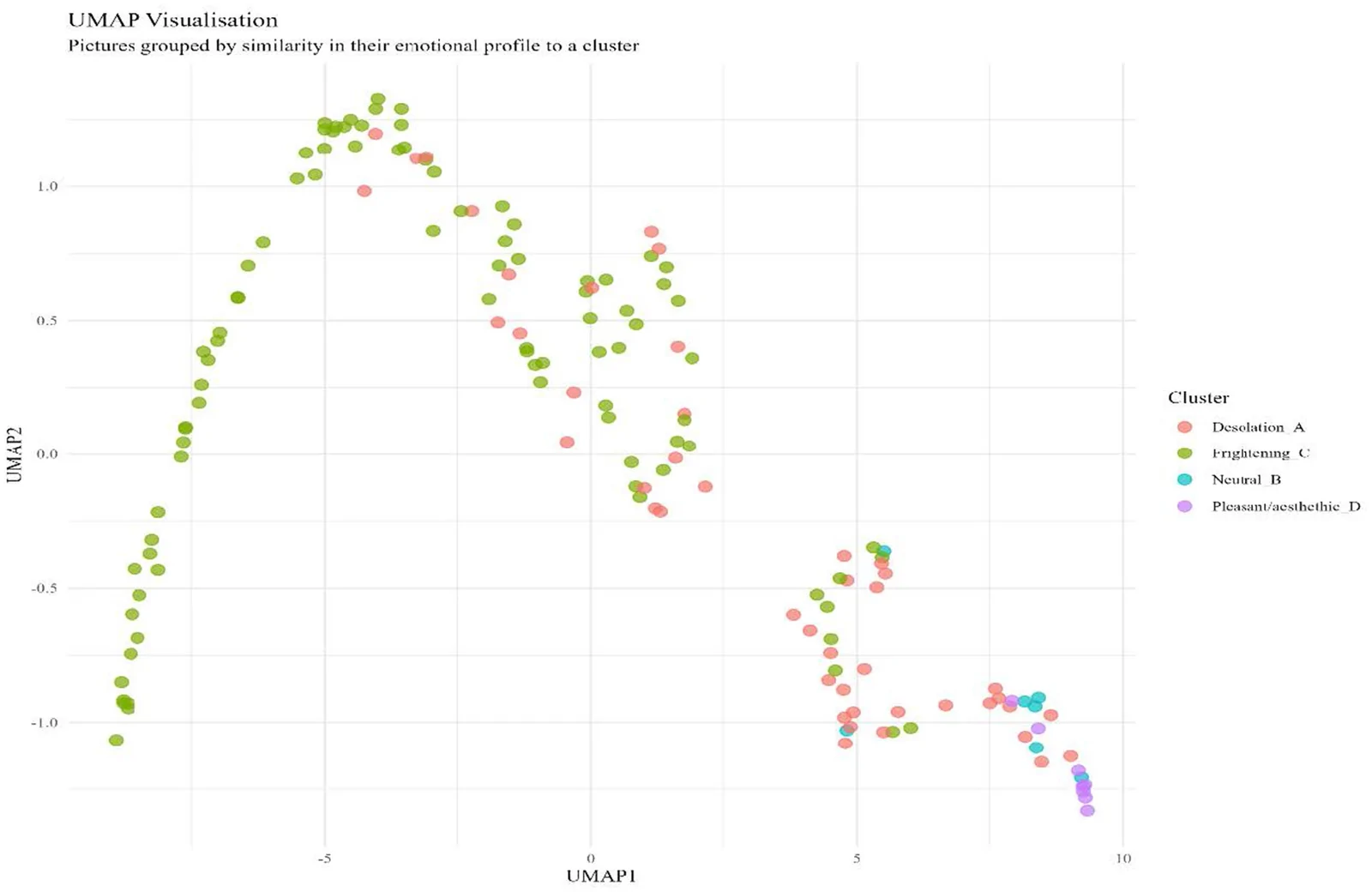
UMAP visualization.

## Discussion

The main objective of the present study was to adapt and validate the Natural Disaster Pictures System (NDPS) for the Spanish population. The results obtained not only confirm the usefulness and reliability of NDPS in the cultural context but also offer deeper insight into perceptions of natural disasters.

The most relevant result of this study was the replication of the emotional structure of the original version of the NDPS. The strong negative association between Valence and Arousal, and the positive relationship of Valence with Dominance and Certainty fully confirm consistency with the original study of (Merlhiot et al. 2018), representing solid evidence of construct validity. This is consistent with previous studies that have used generalized stimulus sets (IAPS, NAPS) to assess the impact of natural disasters. These studies indicated that such stimuli elicit the strongest response in terms of “Arousal” and “Negative Valence” compared with another categories of stimuli (Libkuman et al., 2007; Riegel et al., 2016).

This result was reinforced by the MLM analysis, which validated the applicability of the original classification in four emotional clusters-“Frightening,” “Desolation,” “Pleasant/Aesthetic” and “Neutral”-in the Spanish sample. *Post-hoc* comparisons showed that these categories are mostly statistically distinguishable from one another, confirming that the emotional structure proposed by the databases' authors is cross-culturally robust.

The analysis of group correlations showed a significant difference in Valence, in the inter-subject ANOVA. We attribute this result mainly to the fact that group B was presented with more negative pictures on average. We observed that the other three dimensions did not show significant differences and the effect sizes remained low. The validity of the instrument is also noteworthy because the variability of response is mainly due to the content of the pictures rather than individual differences among the participants.

An important finding of the present study was the underlying one-dimensionality in the data. Both PCA-where the first component explained 97.9% of the variance-and the EFA-a single-factor solution-point to the existence of a common latent construct. Although the profiles of the four clusters are different, this result suggests that, in the context of natural disasters, the dimensions of Valence, Arousal, Dominance and Certainty do not operate independently but rather collapse into an overall assessment of the situation.

This one-dimensionality does not invalidate the four-cluster structure. The exploratory cluster analysis, although suggesting an optimal classification of *k* = 2 groups (probably separating high- and low-impact stimuli), showed in the HCA dendrogram that a division into four groups was structurally plausible. Moreover, advanced visualization with UMAP illustrated this result because it showed a clear separation between the two groups (high *vs*. low impact). However, within each group, subgroups could be distinguished that corresponded to the four original clusters.

These findings are supported by MLMs with substantial effect sizes, which demonstrate that although the dimensions are related, the classification explains around 60% of the variance in emotional response for clusters and more than 75% for classes. This confirms that the instrument retains a high level of discriminatory power.

The Random Forest classifier, although it achieved an accuracy of 75.7% showed a reduced sensitivity for the “Desolation” cluster, frequently confusing it with “Frightening.” Dominance was the dimension that provided the most discriminative information for separating emotional profiles, especially between clusters with medium to high perceived control (“Neutral” and “Pleasant/Aesthetic”), which create a sense of security and calm, and clusters with low perceived control (“Frightening” and “Desolation”), which evoke feeling of helplessness or threat. These results are consistent with the Appraisal Tendency Framework (ATF) discussed in the following paragraph.

Both the *Machine Learning* model and the UMAP visualization revealed a significant overlap between the “Frightening” (fear) and “Desolation” (sadness) clusters. It suggests that, when faced with pictures of natural disasters, the responses of fear and sadness are perceptually very similar and difficult for participants to separate. This result is theoretically important and consistent with findings from other picture databases such as GAPED (Dan-Glauser and Scherer, 2011), which also showed this overlap between fear and sadness in threatening contexts. Based on Scherer's Component Process Model (CPM) (Scherer et al., 2001), which underpins the structure of the GAPED, both emotions share the primary valence of “obstruction of goals,” resulting in a convergent score on the negative valence dimension. Likewise, in the dimensional model of affect (Bradley and Lang, 1994; Russell, 2003), this overlap is explained because both, sadness and fear are located in similar quadrant in the affective space, characterized by markedly low levels of valence. Furthermore, according to the Appraisal Tendency Framework (ATF) (Smith and Ellsworth, 1985; Lerner and Keltner, 2001, 2000) fear and sadness differ in the level of perceived certainty, but both emotions converge in a perception of low dominance and low situational control when a person is face with overwhelming external events. For example, uncertainty regarding an imminent danger (fear) *vs*. certainty of a loss that has already happened (sadness).

Finally, we explored Merlhiot et al. (2018) theoretical classes to give the results greater conceptual validity. The results confirmed that this theoretical categorization is highly robust (*p* < 0.001) demonstrating that the semantic content of pictures influence emotion response. In addition, our study using MLM allowed us to distinguish more effectively between Desolation/loss and Desolation/threat, observing that loss generates a significantly more negative impact than threat. This result may be due, first and foremost, to the specific characteristics of the Spanish sample, which may differ culturally from French sample. This results may be also due to our more in-depth analysis of the data than that conducted by Merlhiot et al. (2018) in their study. From a theoretical perspective, the fact that the loss elicits a significantly more negative emotional response than the threat categories is in line with Lazarus and Folkman's (2026) Transactional Model of Stress and Coping. According to this model, the emotional impact is determined by the primary assessment of the event: threat is assessed as a possibility of future damage which allows coping strategies, while loss represents damage that has already occurred and, therefore, is irreversible. This suggest that the instrument has the necessary discriminatory power to make a phenomenological distinction between threat and loss, which has important implications for research into decision-making and risk perception.

### Limitations and future lines of research

The study is not without limitations that need to be discussed. The most important limitation is the sample size (*N* = 73), that, although adequate for assessing the stability of correlations and the factorial evaluation, the number of participants is smaller than that used in the original study (*N* = 120) However, it should be noted that although the sample size is moderate, the high communalities observed ensure the recovery of the factors and the stability of the resulting structure. The recruitment strategy followed using LinkedIn may have introduced a selection in the sample, as the platforms tends to group together profiles that match the characteristics of the WEIRD (*Western, Educated, Industrialized, Rich, and Democratic*) samples. Another limitation is that the cross-sectional design did not allow to assess test-retest reliability. Moreover, as the emotional assessment was conducted through self-report (SAM), response bias is possible. Finally, even though the study was designed to recruit participants who had experienced natural disasters, the subsample collected was too small-8 participants-to make possible intergroup comparisons due to the lack of power and robustness necessary to draw valid conclusions. In this sense, the direct applicability of this adaptation to other specific populations or cultural contexts should be approach with caution.

The implications suggested below are not conclusions directly supported by the analyses in this study but suggest ideas for future research. For example, a line for future research will be the characterization of emotional profiles in people exposed to traumatic events, such as survivors of natural disaster. To this end, the use emotional induction protocols such as that used by Yang et al. (2004) is interesting. Also, to progress toward a more comprehensive paradigm would require the incorporation of psychophysiological data (e.g., skin conductance, electrocardiography) in experimental contexts. This will ensure that the instrument can identify specific reactivity patterns, like stress, strengthening the ecological validity of the findings. In the cognitive domain, the NDPS might have multiple applications providing a standardized framework for investigating the perception of risk in the face of environmental or psychosocial threats, and for analyzing how this influence decision-making in situations of high uncertainty by introducing the “Certainty” dimension. In particular, this dimension will allow to explore the temporal dynamics of emotion processing when a person is coping with a catastrophe. Future research should investigate in depth whether, as the Appraisal Tendency Framework (ATF) (Smith and Ellsworth, 1985; Lerner and Keltner, 2001, 2000) suggests, Certainty improves the discrimination of emotional profiles in samples with different levels of exposure to natural disasters.

## Conclusions

To conclude, this study has shown that the Natural Disaster Picture System (NDPS) is a reliable and valid tool for use in the Spanish population, with great potential in clinical and experimental research contexts.

The implications are significant for understanding the perception of natural disasters, since the data support a hierarchical assessment model which an initial overall assessment of the situation (high *vs*. low impact/good *vs*. bad) is made, which is reflected by the single factor, while the dimensions-Valence, Arousal, Dominance, and Certainty- would play a role in refining this initial assessment into more specific categories, giving rise to the emotional profile of each cluster.

## Data Availability

The original contributions presented in the study are included in the article/Supplementary material; further inquiries can be directed to the corresponding author.
