# Supplementary material for: Adaptation and psychometric evaluation of the Natural Disasters Picture System (NDPS) to the Spanish population
Source: Front Psychol. 2026 Sep 16;17:1911152. doi: 10.3389/fpsyg.2026.1911152 (PMC13624170; doi:10.3389/fpsyg.2026.1911152)
Supplement: Supplementary file 1 [file Supplementary_File_1.doc]

**Appendix 1**

**Table 1**. Sociodemographic Homogenity Analysis.

| **Variable** | **Group A**  **(n = 36)** | **Group B**  **(n = 37)** | **Statistical Test** | **p-value** |
| --- | --- | --- | --- | --- |
| Age (Mean$\pm$SD) | 34.6 (8.2) | 39.7 (13.3) | t(71) = -1.97 | .053 |
| Gender (f/m/*) | 27 / 8 / 1 | 26 / 9 / 2* | $x^{2}$(3) = 1.06 | .786 |

*Note*. * Includes minority categories detected in the sample (Non-Binary, Prefer not to say).

**Figure 1**. Illustration of the sequence in the emotional assessment task*.*

 *Note*. Assessment process carried out by the participants.
