# Supplementary material for: Adaptation and psychometric evaluation of the Natural Disasters Picture System (NDPS) to the Spanish population
Source: Front Psychol. 2026 Sep 16;17:1911152. doi: 10.3389/fpsyg.2026.1911152 (PMC13624170; doi:10.3389/fpsyg.2026.1911152)
Supplement: Supplementary file 2 [file Supplementary_File_2.doc]

**Appendix 2**

**Table 1**. Statistical descriptive per picture.

| **Nº** | **Picture** | **Main Category** | **Valence**  **Mean (SD)** | **Arousal Mean (SD)** | **Dominance**  **Mean (SD)** | **Certainty Mean (SD)** | **Emotion** | **Cluster** | **Class** |
| --- | --- | --- | --- | --- | --- | --- | --- | --- | --- |
| 1 |  | Avalanche | 5.08 (1.84) | 4.83 (1.61) | 4.72 (2.19) | 4.92 (1.92) | Neutral / Interest | Neutral (B) | Neutral (III) |
| 2 |  | Avalanche | 4.50 (1.50) | 5.00 (1.59) | 5.28 (1.34) | 4.61 (1.32) | Neutral / Interest | Neutral (B) | Neutral (III) |
| 3 |  | Earthquake | 2.36 (0.83) | 6.61 (1.76) | 3.31 (1.43) | 3.14 (1.36) | Fear | Frightening (C) | Aftermath / Rubble (VII) |
| 4 |  | Earthquake | 2.47 (1.11) | 7.22 (1.05) | 2.78 (1.12) | 3.11 (1.63) | Fear | Frightening (C) | Aftermath / Rubble (VII) |
| 5 |  | Earthquake | 2.86 (0.96) | 6.22 (1.46) | 3.69 (1.17) | 3.78 (1.24) | Sadness | Desolation (A) | Desolation / loss (II) |
| 6 |  | Earthquake | 2.39 (0.90) | 7.22 (1.12) | 3.00 (1.47) | 2.69 (1.17) | Fear | Frightening (C) | Aftermath / Rubble (VII) |
| 7 |  | Floods | 3.14 (1.48) | 6.47 (2.05) | 2.69 (1.19) | 2.86 (1.25) | Sadness | Desolation (A) | Desolation / loss (II) |
| 8 |  | Floods | 2.36 (0.93) | 7.11 (1.21) | 2.50 (0.94) | 2.64 (1.07) | Fear | Frightening (C) | Aftermath / Rubble (VII) |
| 9 |  | Floods | 2.67 (1.04) | 6.61 (1.99) | 3.25 (1.38) | 3.33 (1.43) | Sadness | Desolation (A) | Desolation / loss (II) |
| 10 |  | Fire | 1.75 (0.87) | 8.14 (0.76) | 2.17 (1.28) | 2.56 (1.32) | Fear | Frightening (C) | Highly shocking (V) |
| 11 |  | Fire | 2.28 (0.97) | 7.11 (1.09) | 2.97 (1.70) | 3.22 (1.49) | Fear | Frightening (C) | Shocking (IV) |
| 12 |  | Fire | 2.64 (0.93) | 6.39 (2.45) | 3.36 (1.59) | 2.75 (1.34) | Fear | Frightening (C) | Shocking (IV) |
| 13 |  | Hurricane | 2.47 (1.06) | 7.00 (1.51) | 2.83 (1.06) | 3.00 (1.33) | Fear | Frightening (C) | Aftermath / Rubble (VII) |
| 14 |  | Hurricane | 2.50 (0.77) | 7.06 (0.83) | 3.17 (1.08) | 3.06 (1.17) | Fear | Frightening (C) | Aftermath / Rubble (VII) |
| 15 |  | Hurricane | 2.50 (1.06) | 6.86 (1.31) | 3.06 (1.24) | 3.22 (1.64) | Fear | Frightening (C) | Aftermath / Rubble (VII) |
| 16 |  | Hurricane | 2.69 (1.72) | 7.22 (0.80) | 2.47 (0.91) | 2.72 (1.50) | Fear | Frightening (C) | Aftermath / Rubble (VII) |
| 17 |  | Landslide | 2.89 (1.14) | 6.69 (1.19) | 3.56 (1.78) | 3.42 (1.54) | Fear | Frightening (C) | Aftermath / Rubble (VII) |
| 18 |  | Landslide | 3.33 (0.76) | 6.64 (0.80) | 3.69 (1.12) | 3.39 (1.55) | Sadness | Desolation (A) | Desolation / loss (II) |
| 19 |  |  |  |  |  |  |  |  |  |
| 20 |  | Tornado | 3.33 (1.33) | 6.25 (1.56) | 3.86 (1.68) | 3.58 (1.61) | Fear | Frightening (C) | Shocking (IV) |
| 21 |  | Tornado | 3.06 (1.07) | 6.61 (1.42) | 3.36 (1.40) | 3.08 (1.38) | Fear | Frightening (C) | Shocking (IV) |
| 22 |  | Tornado | 3.17 (1.00) | 6.75 (1.23) | 3.50 (1.32) | 3.00 (1.41) | Fear | Frightening (C) | Shocking (IV) |
| 23 |  | Tsunami | 2.03 (0.74) | 7.94 (0.83) | 2.11 (1.01) | 2.06 (1.24) | Fear | Frightening (C) | Highly shocking (V) |
| 24 |  | Tsunami | 2.06 (0.83) | 7.58 (1.11) | 2.50 (1.46) | 1.83 (1.18) | Fear | Frightening (C) | Aftermath / Rubble (VII) |
| 25 |  | Tsunami | 2.56 (1.13) | 6.78 (1.51) | 3.25 (1.18) | 3.22 (1.31) | Fear | Frightening (C) | Aftermath / Rubble (VII) |
| 26 |  | Tsunami | 2.25 (0.91) | 7.50 (1.03) | 2.42 (1.27) | 2.39 (0.99) | Fear | Frightening (C) | Aftermath / Rubble (VII) |
| 27 |  | Typhoon | 3.94 (0.89) | 5.47 (1.21) | 3.94 (1.84) | 4.06 (0.95) | Sadness | Desolation (A) | Desolation / loss (II) |
| 28 |  | Typhoon | 3.36 (0.72) | 6.25 (0.81) | 4.28 (1.50) | 3.64 (1.51) | Sadness | Desolation (A) | Desolation / loss (II) |
| 29 |  | Typhoon | 2.44 (1.00) | 6.56 (1.50) | 3.31 (1.45) | 3.17 (1.50) | Fear | Frightening (C) | Aftermath / Rubble (VII) |
| 30 |  | Eruption | 4.72 (1.65) | 5.14 (1.79) | 5.33 (1.76) | 4.64 (1.61) | Sadness | Desolation (A) | Desolation / loss (II) |
| 31 |  | Eruption | 5.19 (1.49) | 4.36 (1.93) | 4.50 (2.13) | 4.89 (1.63) | Fear | Frightening (C) | Volcanic eruption (VIII) |
| 32 |  | Eruption | 5.11 (1.88) | 4.72 (1.43) | 5.03 (2.32) | 5.11 (1.86) | Sadness | Desolation (A) | Desolation / threat (I) |
| 33 |  | Eruption | 4.78 (1.85) | 4.56 (1.56) | 5.56 (1.68) | 5.19 (1.92) | Sadness | Desolation (A) | Desolation / threat (I) |
| 34 |  | Eruption | 4.36 (1.44) | 4.83 (1.78) | 5.19 (1.62) | 4.39 (1.46) | Sadness | Desolation (A) | Desolation / loss (II) |
| 35 |  | Eruption | 4.61 (2.05) | 4.39 (1.55) | 5.58 (1.65) | 5.28 (1.45) | Fear | Frightening (C) | Volcanic eruption (VIII) |
| 36 |  | Eruption | 3.69 (1.88) | 5.39 (1.44) | 4.97 (1.50) | 4.33 (1.71) | Sadness | Desolation (A) | Desolation / loss (II) |
| 37 |  | Eruption | 4.69 (1.41) | 5.28 (1.37) | 4.89 (1.53) | 4.61 (1.57) | Sadness | Desolation (A) | Desolation / loss (II) |
| 38 |  | Eruption | 2.39 (0.77) | 6.89 (0.89) | 3.06 (1.35) | 3.06 (1.57) | Fear | Frightening (C) | Shocking (IV) |
| 39 |  | Lava Flow | 2.36 (0.76) | 7.56 (0.91) | 2.81 (1.49) | 2.78 (1.20) | Fear | Frightening (C) | Highly shocking (V) |
| 40 |  | Pyroclastic Flow | 3.36 (1.22) | 5.72 (1.77) | 4.78 (1.84) | 4.22 (1.40) | Sadness | Desolation (A) | Desolation / loss (II) |
| 41 |  | Tephra falls | 3.97 (0.81) | 5.58 (1.18) | 4.53 (1.61) | 4.22 (1.55) | Sadness | Desolation (A) | Desolation / loss (II) |
| 42 |  | Eruption | 6.17 (2.34) | 3.03 (1.40) | 6.92 (1.27) | 6.14 (2.31) | Neutral / Interest | Neutral (B) | Neutral (III) |
| 43 |  | Fumarole | 4.61 (1.08) | 5.03 (1.92) | 5.39 (1.59) | 4.69 (1.51) | Sadness | Desolation (A) | Desolation / threat (I) |
| 44 |  | Acid Lake | 6.44 (1.84) | 3.64 (1.84) | 6.58 (1.84) | 6.89 (1.56) | Sadness | Desolation (A) | Desolation / threat (I) |
| 45 |  | Fumarole | 3.81 (0.98) | 5.42 (1.16) | 5.08 (1.44) | 4.75 (1.84) | Sadness | Desolation (A) | Desolation / loss (II) |
| 46 |  | Geyser | 4.17 (1.65) | 5.14 (1.25) | 5.33 (1.74) | 4.92 (1.34) | Sadness | Desolation (A) | Desolation / threat (I) |
| 47 |  | Geyser | 6.25 (1.61) | 3.56 (1.61) | 5.69 (2.28) | 5.89 (2.08) | Sadness | Desolation (A) | Desolation / threat (I) |
| 48 |  | Lahar | 3.42 (1.13) | 5.89 (1.28) | 3.89 (1.79) | 3.86 (1.57) | Fear | Frightening (C) | Aftermath / Rubble (VII) |
| 49 |  | Lahar | 3.69 (1.12) | 5.17 (1.58) | 5.61 (1.57) | 5.06 (1.55) | Sadness | Desolation (A) | Desolation / threat (I) |
| 50 |  | Lahar | 3.53 (1.25) | 5.89 (1.30) | 4.83 (1.78) | 4.39 (1.98) | Sadness | Desolation (A) | Desolation / loss (II) |
| 51 |  | Lahar | 2.97 (0.88) | 6.69 (1.53) | 3.36 (1.40) | 3.36 (1.33) | Sadness | Desolation (A) | Desolation / loss (II) |
| 52 |  | Lava Flow | 4.08 (1.59) | 5.42 (2.26) | 4.25 (1.98) | 3.94 (1.53) | Fear | Frightening (C) | Shocking (IV) |
| 53 |  | Lava Flow | 3.00 (1.07) | 6.19 (1.56) | 3.69 (1.56) | 3.92 (1.52) | Fear | Frightening (C) | Shocking (IV) |
| 54 |  | Lava Flow | 3.25 (1.18) | 5.72 (1.78) | 4.03 (1.83) | 3.97 (1.28) | Fear | Frightening (C) | Shocking (IV) |
| 55 |  | Lava Flow | 3.42 (1.20) | 5.92 (1.40) | 4.28 (1.91) | 4.28 (1.77) | Fear | Frightening (C) | Aftermath / Rubble (VII) |
| 56 |  | Lava Flow | 3.44 (1.08) | 6.11 (1.17) | 4.53 (1.50) | 4.14 (1.25) | Fear | Frightening (C) | Aftermath / Rubble (VII) |
| 57 |  | Lava Flow | 2.39 (1.32) | 7.53 (1.30) | 2.39 (1.38) | 2.61 (1.36) | Fear | Frightening (C) | Volcanic eruption (VIII) |
| 58 |  | Lava Flow | 3.81 (1.37) | 5.97 (1.40) | 4.06 (1.80) | 4.08 (1.59) | Fear | Frightening (C) | Volcanic eruption (VIII) |
| 59 |  | Lava Flow | 3.36 (1.36) | 5.67 (1.60) | 3.50 (1.65) | 4.00 (1.80) | Fear | Frightening (C) | Aftermath / Rubble (VII) |
| 60 |  | Tephra falls | 4.11 (1.60) | 5.11 (1.47) | 5.00 (1.57) | 4.83 (1.46) | Sadness | Desolation (A) | Desolation / loss (II) |
| 61 |  | Tephra falls | 3.94 (1.39) | 5.17 (1.36) | 4.36 (1.64) | 4.36 (1.73) | Sadness | Desolation (A) | Desolation / loss (II) |
| 62 |  | Tephra falls | 4.47 (1.80) | 4.67 (1.51) | 5.44 (1.54) | 5.14 (1.61) | Sadness | Desolation (A) | Desolation / loss (II) |
| 63 |  | Tephra falls | 3.19 (1.17) | 6.44 (1.23) | 4.25 (1.52) | 4.42 (1.71) | Sadness | Desolation (A) | Desolation / loss (II) |
| 64 |  | Tephra falls | 2.89 (1.19) | 6.78 (1.27) | 3.17 (1.36) | 2.94 (1.17) | Fear | Frightening (C) | Aftermath / Rubble (VII) |
| 65 |  | Pyroclastic Flow | 4.19 (1.17) | 4.69 (1.88) | 5.19 (1.60) | 4.69 (1.21) | Sadness | Desolation (A) | Desolation / loss (II) |
| 66 |  | Pyroclastic Flow | 3.83 (1.65) | 5.28 (1.14) | 5.25 (1.27) | 4.22 (1.73) | Sadness | Desolation (A) | Desolation / loss (II) |
| 67 |  | Pyroclastic Flow | 4.42 (1.50) | 4.86 (1.61) | 5.31 (1.69) | 4.83 (1.56) | Sadness | Desolation (A) | Desolation / loss (II) |
| 68 |  | Pyroclastic Flow | 4.08 (1.52) | 5.53 (1.46) | 4.42 (1.87) | 3.89 (1.47) | Fear | Frightening (C) | Volcanic eruption (VIII) |
| 69 |  | Eruption | 5.58 (1.87) | 3.72 (1.65) | 6.17 (1.38) | 5.25 (2.21) | Sadness | Desolation (A) | Desolation / loss (II) |
| 70 |  | Eruption | 5.03 (1.36) | 4.69 (1.51) | 5.00 (1.85) | 4.72 (1.67) | Sadness | Desolation (A) | Desolation / threat (I) |
| 71 |  | Eruption | 5.00 (1.59) | 4.89 (1.28) | 4.58 (1.83) | 4.64 (1.66) | Sadness | Desolation (A) | Desolation / loss (II) |
| 72 |  | Eruption | 3.36 (0.99) | 5.81 (1.35) | 4.42 (1.71) | 4.14 (1.64) | Fear | Frightening (C) | Volcanic eruption (VIII) |
| 73 |  | Eruption | 3.89 (1.59) | 5.65 (1.72) | 4.54 (1.91) | 4.43 (1.50) | Fear | Frightening (C) | Volcanic eruption (VIII) |
| 74 |  | Lava Flow | 4.62 (1.36) | 5.11 (1.56) | 5.00 (1.80) | 5.16 (1.76) | Sadness | Desolation (A) | Desolation / loss (II) |
| 75 |  | Eruption | 4.19 (1.66) | 5.03 (1.42) | 5.08 (1.83) | 5.08 (1.74) | Fear | Frightening (C) | Volcanic eruption (VIII) |
| 76 |  | Eruption | 4.35 (1.65) | 5.46 (1.64) | 4.24 (2.09) | 4.38 (1.50) | Fear | Frightening (C) | Volcanic eruption (VIII) |
| 77 |  | Eruption | 3.38 (1.53) | 6.00 (1.39) | 4.35 (1.75) | 3.84 (1.38) | Fear | Frightening (C) | Volcanic eruption (VIII) |
| 78 |  | Avalanche | 4.14 (1.57) | 5.78 (1.69) | 4.05 (1.68) | 4.08 (1.64) | Fear | Frightening (C) | Volcanic eruption (VIII) |
| 79 |  | Avalanche | 4.24 (1.53) | 5.51 (1.64) | 4.11 (1.90) | 4.05 (1.61) | Sadness | Desolation (A) | Desolation / loss (II) |
| 80 |  | Earthquake | 3.03 (0.96) | 6.27 (1.22) | 3.92 (1.40) | 3.76 (1.48) | Fear | Frightening (C) | Aftermath / Rubble (VII) |
| 81 |  | Earthquake | 3.16 (1.09) | 6.49 (1.22) | 3.49 (1.45) | 3.41 (1.36) | Fear | Frightening (C) | Aftermath / Rubble (VII) |
| 82 |  | Fire | 1.97 (1.04) | 7.57 (1.41) | 2.59 (1.50) | 2.73 (1.41) | Fear | Frightening (C) | Shocking (IV) |
| 83 |  | Fire | 3.57 (1.19) | 5.84 (1.64) | 4.62 (1.66) | 4.00 (1.58) | Sadness | Desolation (A) | Desolation / loss (II) |
| 84 |  | Fire | 1.95 (0.88) | 7.65 (1.06) | 2.16 (1.21) | 2.43 (1.37) | Fear | Frightening (C) | Highly shocking (V) |
| 85 |  | Fire | 2.43 (0.90) | 7.35 (0.92) | 3.32 (1.42) | 2.97 (1.24) | Fear | Frightening (C) | Aftermath / Rubble (VII) |
| 86 |  | Fire | 2.00 (0.91) | 7.49 (1.22) | 2.57 (1.34) | 2.86 (1.48) | Fear | Frightening (C) | Highly shocking (V) |
| 87 |  | Fire | 3.32 (1.18) | 6.46 (1.48) | 3.86 (1.57) | 3.38 (1.52) | Fear | Frightening (C) | Shocking (IV) |
| 88 |  | Floods | 2.49 (0.90) | 7.24 (1.04) | 2.76 (1.32) | 2.73 (1.43) | Fear | Frightening (C) | Shocking (IV) |
| 89 |  | Floods | 3.05 (1.03) | 6.59 (1.17) | 3.59 (1.28) | 3.30 (1.33) | Fear | Frightening (C) | Shocking (IV) |
| 90 |  | Hurricane | 3.11 (1.66) | 6.86 (1.99) | 3.57 (1.66) | 3.41 (1.61) | Sadness | Desolation (A) | Desolation / loss (II) |
| 91 |  | Tornado | 3.32 (1.11) | 6.38 (1.23) | 3.84 (1.32) | 3.51 (1.26) | Fear | Frightening (C) | Aftermath / Rubble (VII) |
| 92 |  | Tornado | 3.08 (0.95) | 6.41 (1.26) | 4.14 (1.70) | 4.00 (1.56) | Fear | Frightening (C) | Aftermath / Rubble (VII) |
| 93 |  | Landslide | 3.32 (1.43) | 6.24 (1.82) | 3.97 (1.44) | 3.81 (1.29) | Fear | Frightening (C) | Aftermath / Rubble (VII) |
| 94 |  | Landslide | 3.27 (1.15) | 6.41 (1.26) | 3.95 (1.47) | 3.49 (1.19) | Sadness | Desolation (A) | Desolation / loss (II) |
| 95 |  | Tornado | 3.27 (1.10) | 6.43 (1.30) | 3.54 (1.54) | 3.65 (1.46) | Fear | Frightening (C) | Highly shocking (V) |
| 96 |  | Tornado | 3.11 (1.37) | 6.41 (1.21) | 3.81 (1.49) | 3.49 (1.41) | Fear | Frightening (C) | Highly shocking (V) |
| 97 |  | Sea Storm | 5.89 (2.25) | 3.68 (1.87) | 6.59 (1.83) | 6.49 (1.80) | Neutral / Interest | Neutral (B) | Neutral (III) |
| 98 |  | Sea Storm | 4.68 (2.01) | 5.54 (1.80) | 5.05 (2.13) | 4.92 (1.98) | Fear | Frightening (C) | Shocking (IV) |
| 99 |  | Sea Storm | 3.70 (1.00) | 5.81 (1.33) | 4.57 (1.66) | 4.22 (1.38) | Fear | Frightening (C) | Shocking (IV) |
| 100 |  | Lava Flow | 6.11 (2.47) | 3.49 (1.89) | 6.38 (1.89) | 6.43 (1.72) | Sadness | Desolation (A) | Desolation / threat (I) |
| 101 |  | Lava Flow | 5.70 (1.90) | 4.24 (2.02) | 5.81 (2.01) | 5.62 (1.74) | Sadness | Desolation (A) | Desolation / loss (II) |
| 102 |  | Lava Flow | 6.38 (1.91) | 3.70 (1.88) | 6.51 (1.87) | 6.62 (1.78) | Neutral / Interest | Neutral (B) | Neutral (III) |
| 103 |  | Lava Flow | 5.65 (1.78) | 4.70 (1.90) | 5.57 (1.97) | 5.65 (1.90) | Sadness | Desolation (A) | Desolation / threat (I) |
| 104 |  | Lava Flow | 3.19 (1.43) | 6.35 (1.46) | 3.89 (1.76) | 3.89 (1.45) | Fear | Frightening (C) | Shocking (IV) |
| 105 |  | Lava Flow | 3.51 (1.02) | 5.84 (1.61) | 3.70 (1.63) | 3.62 (1.26) | Fear | Frightening (C) | Shocking (IV) |
| 106 |  | Lava Flow | 4.00 (1.37) | 5.43 (1.63) | 4.86 (1.77) | 4.86 (1.73) | Fear | Frightening (C) | Volcanic eruption (VIII) |
| 107 |  | Tephra falls | 3.05 (1.18) | 6.51 (1.28) | 3.65 (1.60) | 3.70 (1.31) | Sadness | Desolation (A) | Desolation / threat (I) |
| 108 |  | Tephra falls | 3.65 (1.06) | 5.97 (1.44) | 3.89 (1.76) | 3.65 (1.32) | Sadness | Desolation (A) | Desolation / loss (II) |
| 109 |  | Tephra falls | 1.78 (0.95) | 8.11 (1.05) | 2.32 (1.55) | 2.49 (1.41) | Fear | Frightening (C) | Victims (VI) |
| 110 |  | Lava Flow | 3.00 (1.15) | 7.03 (1.14) | 3.54 (1.48) | 3.11 (1.22) | Fear | Frightening (C) | Shocking (IV) |
| 111 |  | Tephra falls | 3.59 (1.46) | 5.62 (1.52) | 4.51 (1.52) | 4.51 (1.48) | Sadness | Desolation (A) | Desolation / loss (II) |
| 112 |  | Lahar | 3.03 (1.24) | 6.65 (1.23) | 3.22 (1.51) | 3.38 (1.64) | Fear | Frightening (C) | Shocking (IV) |
| 113 |  | Lava Flow | 2.57 (0.93) | 6.73 (1.02) | 3.70 (1.41) | 3.73 (1.41) | Fear | Frightening (C) | Aftermath / Rubble (VII) |
| 114 |  | Pyroclastic Flow | 3.70 (1.53) | 5.92 (1.34) | 4.19 (1.39) | 3.84 (1.34) | Fear | Frightening (C) | Aftermath / Rubble (VII) |
| 115 |  | Eruption | 4.22 (1.93) | 5.03 (1.80) | 5.00 (1.75) | 4.73 (1.45) | Fear | Frightening (C) | Volcanic eruption (VIII) |
| 116 |  | Eruption | 4.49 (1.35) | 5.73 (1.45) | 4.73 (1.68) | 4.32 (1.75) | Fear | Frightening (C) | Volcanic eruption (VIII) |
| 117 |  | Eruption | 4.86 (1.70) | 5.05 (1.56) | 4.59 (2.25) | 4.95 (1.90) | Fear | Frightening (C) | Volcanic eruption (VIII) |
| 118 |  | Eruption | 2.86 (1.49) | 7.00 (1.45) | 3.11 (1.74) | 3.16 (1.62) | Fear | Frightening (C) | Volcanic eruption (VIII) |
| 119 |  | Fumarole | 4.92 (2.06) | 4.84 (1.79) | 5.70 (1.53) | 5.84 (1.52) | Fear | Frightening (C) | Volcanic eruption (VIII) |
| 120 |  | Lava Flow | 3.95 (1.65) | 5.78 (2.03) | 3.89 (1.73) | 3.59 (1.67) | Fear | Frightening (C) | Shocking (IV) |
| 121 |  | Lava Flow | 3.49 (1.28) | 6.03 (1.52) | 4.30 (1.78) | 3.84 (1.44) | Fear | Frightening (C) | Shocking (IV) |
| 122 |  | Acid Lake | 8.03 (1.14) | 1.97 (0.93) | 7.86 (1.03) | 7.84 (1.09) | Aesthetic emotion | Pleasant / aesthetic (D) | Pleasant / aesthetic (IX) |
| 123 |  | Acid Lake | 7.19 (2.23) | 2.81 (1.88) | 6.97 (1.99) | 7.16 (1.88) | Neutral / Interest | Neutral (B) | Neutral (III) |
| 124 |  | Acid Lake | 6.59 (1.88) | 3.05 (1.82) | 6.73 (1.76) | 6.65 (1.80) | Sadness | Desolation (A) | Desolation / threat (I) |
| 125 |  | Acid Lake | 7.41 (1.26) | 2.59 (1.28) | 7.24 (1.26) | 7.46 (0.99) | Aesthetic emotion | Pleasant / aesthetic (D) | Pleasant / aesthetic (IX) |
| 126 |  | Acid Lake | 7.68 (1.62) | 2.22 (1.27) | 7.49 (1.57) | 7.51 (1.77) | Aesthetic emotion | Pleasant / aesthetic (D) | Pleasant / aesthetic (IX) |
| 127 |  | Lava Flow | 3.86 (1.60) | 5.89 (1.84) | 4.41 (1.98) | 4.57 (1.77) | Fear | Frightening (C) | Shocking (IV) |
| 128 |  | Pyroclastic Flow | 4.24 (1.06) | 5.41 (1.17) | 4.89 (1.54) | 4.78 (1.75) | Fear | Frightening (C) | Volcanic eruption (VIII) |
| 129 |  | Pyroclastic Flow | 3.49 (1.30) | 6.32 (1.42) | 3.51 (1.48) | 3.68 (1.33) | Fear | Frightening (C) | Volcanic eruption (VIII) |
| 130 |  | Eruption | 3.78 (1.62) | 5.38 (2.27) | 4.57 (1.82) | 4.35 (1.74) | Fear | Frightening (C) | Volcanic eruption (VIII) |
| 131 |  | Eruption | 3.76 (1.69) | 5.68 (1.86) | 4.16 (1.94) | 4.05 (1.84) | Fear | Frightening (C) | Volcanic eruption (VIII) |
| 132 |  | Victims | 1.65 (0.92) | 7.78 (1.11) | 2.32 (1.49) | 2.54 (1.37) | Fear | Frightening (C) | Aftermath / Rubble (VII) |
| 133 |  | Victims | 1.89 (1.05) | 7.86 (1.16) | 2.08 (1.16) | 2.30 (1.41) | Fear | Frightening (C) | Victims (VI) |
| 134 |  | Victims | 1.76 (0.93) | 7.86 (1.13) | 2.27 (1.35) | 2.46 (1.41) | Fear | Frightening (C) | Victims (VI) |
| 135 |  | Victims | 1.70 (0.91) | 8.22 (1.13) | 2.32 (1.68) | 2.35 (1.25) | Fear | Frightening (C) | Victims (VI) |
| 136 |  | Victims | 1.84 (0.96) | 7.92 (1.59) | 2.24 (1.26) | 2.57 (1.64) | Fear | Frightening (C) | Victims (VI) |
| 137 |  | Victims | 2.03 (1.04) | 7.51 (1.88) | 2.43 (1.44) | 2.46 (1.41) | Fear | Frightening (C) | Aftermath / Rubble (VII) |
| 138 |  | Victims | 2.32 (1.58) | 7.57 (1.57) | 2.46 (1.71) | 2.38 (1.32) | Fear | Frightening (C) | Aftermath / Rubble (VII) |
| 139 |  | Victims | 2.22 (1.77) | 8.00 (1.05) | 2.59 (1.69) | 2.19 (1.22) | Fear | Frightening (C) | Aftermath / Rubble (VII) |
| 140 |  | Victims | 1.76 (1.01) | 8.00 (1.22) | 2.24 (1.72) | 2.16 (1.38) | Fear | Frightening (C) | Victims (VI) |
| 141 |  | Victims | 2.27 (1.56) | 7.59 (1.76) | 2.65 (1.51) | 2.65 (1.48) | Fear | Frightening (C) | Victims (VI) |
| 142 |  | Victims | 1.51 (0.87) | 8.11 (1.56) | 2.27 (1.39) | 2.35 (1.40) | Fear | Frightening (C) | Victims (VI) |
| 143 |  | Victims | 2.43 (1.42) | 7.51 (1.26) | 2.59 (1.44) | 2.95 (1.61) | Fear | Frightening (C) | Aftermath / Rubble (VII) |
| 144 |  | Victims | 1.70 (0.97) | 7.97 (1.14) | 2.24 (1.36) | 2.22 (1.38) | Fear | Frightening (C) | Victims (VI) |
| 145 |  | Victims | 1.95 (1.10) | 7.70 (1.43) | 2.27 (1.41) | 2.30 (1.39) | Fear | Frightening (C) | Aftermath / Rubble (VII) |
| 146 |  | Victims | 2.38 (1.01) | 7.16 (1.26) | 3.41 (1.57) | 3.43 (1.41) | Fear | Frightening (C) | Aftermath / Rubble (VII) |
| 147 |  | Victims | 2.08 (1.19) | 7.62 (1.72) | 2.38 (1.57) | 2.54 (1.69) | Fear | Frightening (C) | Shocking (IV) |
| 148 |  | Volcano | 5.78 (2.07) | 3.86 (1.81) | 6.16 (1.57) | 6.16 (1.69) | Aesthetic emotion | Pleasant / aesthetic (D) | Pleasant / aesthetic (IX) |
| 149 |  | Volcano | 7.32 (1.20) | 2.62 (1.38) | 7.32 (1.16) | 7.32 (1.16) | Aesthetic emotion | Pleasant / aesthetic (D) | Pleasant / aesthetic (IX) |
| 150 |  | Volcano | 5.81 (1.60) | 3.86 (1.62) | 6.00 (1.72) | 5.65 (1.96) | Sadness | Desolation (A) | Desolation / threat (I) |
| 151 |  | Volcano | 6.27 (1.69) | 3.43 (1.34) | 6.43 (1.66) | 6.46 (1.59) | Aesthetic emotion | Pleasant / aesthetic (D) | Pleasant / aesthetic (IX) |
| 152 |  | Volcano | 6.49 (1.46) | 3.24 (1.40) | 6.51 (1.45) | 6.16 (1.72) | Neutral / Interest | Neutral (B) | Neutral (III) |
| 153 |  | Volcano | 7.76 (1.57) | 2.22 (1.11) | 7.73 (1.04) | 7.81 (1.02) | Aesthetic emotion | Pleasant / aesthetic (D) | Pleasant / aesthetic (IX) |
| 154 |  | Volcano | 7.30 (1.18) | 2.84 (1.38) | 7.24 (1.16) | 6.95 (1.73) | Aesthetic emotion | Pleasant / aesthetic (D) | Pleasant / aesthetic (IX) |
| 155 |  | Volcano | 6.97 (1.46) | 3.00 (1.67) | 6.84 (1.72) | 7.05 (1.13) | Sadness | Desolation (A) | Desolation / threat (I) |

*Note*. Reprinted with permission by Merlhiot et al. (2018), under licence restricting use to non-profit scientific applications conducted by researchers officially affiliated with recognised universities; for further information, please see the link: <https://ndps.lapsco.fr/>.
