# Supplementary material for: Adaptation and psychometric evaluation of the Natural Disasters Picture System (NDPS) to the Spanish population
Source: Front Psychol. 2026 Sep 16;17:1911152. doi: 10.3389/fpsyg.2026.1911152 (PMC13624170; doi:10.3389/fpsyg.2026.1911152)
Supplement: Supplementary file 3 [file Supplementary_File_3.doc]

**Appendix 3**

**Figure 1.** Boxplot of the inter-subjects ANOVA for groups A vs B

*Note*. Comparison of mean by groups (n_A_ = 36, n_B_ = 37) in the dimensions evaluated. The boxes indicate the median and interquartile range; the dots represent the individual scores of the participants. There is a clear overlap in Arousal, Dominance and Certainty, while Valence shows a significant shift between groups A and B (p = .012).
