# Supplementary material for: Adaptation and psychometric evaluation of the Natural Disasters Picture System (NDPS) to the Spanish population
Source: Front Psychol. 2026 Sep 16;17:1911152. doi: 10.3389/fpsyg.2026.1911152 (PMC13624170; doi:10.3389/fpsyg.2026.1911152)
Supplement: Supplementary file 4 [file Supplementary_File_4.doc]

**Appendix 4**

**Figure 1.** Elbow Method.

*Note*. The inflection point (“elbow”) where the curve flattens significantly occurs at cluster k = 3 or k = 4, indicating that adding more clusters after this point no longer improves the model.

**Figure 2**. Silhouette Method.

*Note*. The highest value in the silhouette coefficient is achieved with k = 2 or k = 3, suggesting maximum splitting in terms of group cohesion.

**Figure 3**. K-Means (*k* = 2).

*Note*. Each point represents as picture, coloured according to the cluster to which it was assigned by the analysis of the Spanish sample. The solution archived a Total Within – Cluster Sum of Squares (WSS) of 223.91.

**Figure 4**. K-Means (*k* = 3).

*Note*. Each point represents as picture, coloured according to the cluster to which it was assigned by the analysis of the Spanish sample. The solution archived a Total Within – Cluster Sum of Squares (WSS) of 95.70.

**Figure 5**. K-Means (*k* = 4).

*Note*. Each point represents as picture, coloured according to the cluster to which it was assigned by the analysis of the Spanish sample. The solution archived a Total Within – Cluster Sum of Squares (WSS) of 59.11.

**Figure 6**. K-Means (*k* = 5).

*Note*. Each point represents as picture, coloured according to the cluster to which it was assigned by the analysis of the Spanish sample. The solution archived a Total Within – Cluster Sum of Squares (WSS) of 42.63.

**Figure 7**. PCA sedimentation scree plot.

*Note*. The height of the bars indicates the value of each component, showing a sharp drop after the first component, which captures most of the information.

**Figure 8**. Biplot of pictures and dimensions (PCA).

*Note*. The biplot shows the 152 pictures and the four dimensions projected onto the plane defined by Dimension 1 (97.9% variance) and Dimension 2 (1.1% variance).

**Figure 9**. Diagram of the factorial model (EFA).

*Note*. Representation of the only latent factor extracted (ML1) from the EFA. The negative loading of the Arousal variable (-0.90) is highlighted, while Dominance, Certainty, and Valence have positive loading (0.90).
