# Supplementary material for: Adaptation and psychometric evaluation of the Natural Disasters Picture System (NDPS) to the Spanish population
Source: Front Psychol. 2026 Sep 16;17:1911152. doi: 10.3389/fpsyg.2026.1911152 (PMC13624170; doi:10.3389/fpsyg.2026.1911152)
Supplement: Supplementary file 5 [file Supplementary_File_5.doc]

**Appendix 5**

**Table 1**

*Normative Factor Scores of Affective Reactivity for the NDPS visual stimuli*

| **Picture** | **Mean** |
| --- | --- |
| img122 | 2,770 |
| img153 | 2,648 |
| img126 | 2,547 |
| img125 | 2,369 |
| img149 | 2,340 |
| img154 | 2,200 |
| img123 | 2,192 |
| img155 | 2,076 |
| img124 | 1,924 |
| img042 | 1,811 |
| img044 | 1,777 |
| img152 | 1,741 |
| img151 | 1,707 |
| img102 | 1,693 |
| img100 | 1,659 |
| img097 | 1,625 |
| img047 | 1,459 |
| img148 | 1,439 |
| img150 | 1,326 |
| img069 | 1,286 |
| img101 | 1,182 |
| img103 | 1,026 |
| img119 | 0,951 |
| img035 | 0,908 |
| img033 | 0,867 |
| img032 | 0,774 |
| img062 | 0,773 |
| img031 | 0,758 |
| img070 | 0,696 |
| img001 | 0,661 |
| img067 | 0,640 |
| img074 | 0,617 |
| img065 | 0,612 |
| img043 | 0,610 |
| img075 | 0,579 |
| img030 | 0,577 |
| img002 | 0,571 |
| img071 | 0,565 |
| img117 | 0,563 |
| img046 | 0,556 |
| img049 | 0,554 |
| img034 | 0,543 |
| img115 | 0,507 |
| img060 | 0,491 |
| img098 | 0,477 |
| img037 | 0,467 |
| img128 | 0,407 |
| img106 | 0,380 |
| img045 | 0,371 |
| img066 | 0,339 |
| img061 | 0,274 |
| img036 | 0,271 |
| img116 | 0,248 |
| img076 | 0,237 |
| img130 | 0,229 |
| img073 | 0,184 |
| img041 | 0,171 |
| img111 | 0,163 |
| img052 | 0,136 |
| img079 | 0,131 |
| img068 | 0,123 |
| img127 | 0,122 |
| img050 | 0,111 |
| img040 | 0,094 |
| img099 | 0,083 |
| img027 | 0,079 |
| img078 | 0,045 |
| img131 | 0,035 |
| img083 | 0,028 |
| img072 | 0,004 |
| img055 | -0,013 |
| img058 | -0,046 |
| img056 | -0,047 |
| img114 | -0,070 |
| img054 | -0,077 |
| img120 | -0,092 |
| img077 | -0,108 |
| img121 | -0,109 |
| img059 | -0,123 |
| img048 | -0,139 |
| img063 | -0,156 |
| img108 | -0,169 |
| img105 | -0,185 |
| img028 | -0,221 |
| img093 | -0,239 |
| img092 | -0,253 |
| img104 | -0,281 |
| img053 | -0,290 |
| img020 | -0,297 |
| img080 | -0,302 |
| img129 | -0,331 |
| img005 | -0,340 |
| img091 | -0,347 |
| img094 | -0,350 |
| img087 | -0,388 |
| img095 | -0,388 |
| img096 | -0,391 |
| img107 | -0,411 |
| img018 | -0,457 |
| img081 | -0,468 |
| img089 | -0,513 |
| img113 | -0,516 |
| img017 | -0,544 |
| img090 | -0,559 |
| img051 | -0,572 |
| img112 | -0,572 |
| img021 | -0,590 |
| img022 | -0,606 |
| img009 | -0,612 |
| img012 | -0,647 |
| img029 | -0,648 |
| img110 | -0,673 |
| img003 | -0,678 |
| img007 | -0,684 |
| img025 | -0,689 |
| img064 | -0,709 |
| img118 | -0,738 |
| img015 | -0,746 |
| img146 | -0,749 |
| img038 | -0,798 |
| img014 | -0,810 |
| img011 | -0,851 |
| img013 | -0,859 |
| img085 | -0,886 |
| img004 | -0,904 |
| img006 | -0,957 |
| img088 | -0,980 |
| img016 | -0,991 |
| img008 | -1,017 |
| img143 | -1,041 |
| img039 | -1,060 |
| img086 | -1,108 |
| img141 | -1,128 |
| img057 | -1,141 |
| img082 | -1,153 |
| img026 | -1,188 |
| img138 | -1,191 |
| img137 | -1,205 |
| img147 | -1,219 |
| img084 | -1,296 |
| img145 | -1,318 |
| img024 | -1,323 |
| img132 | -1,325 |
| img139 | -1,330 |
| img136 | -1,342 |
| img134 | -1,354 |
| img133 | -1,394 |
| img109 | -1,400 |
| img010 | -1,423 |
| img023 | -1,437 |
| img144 | -1,437 |
| img140 | -1,447 |
| img135 | -1,463 |
| img142 | -1,468 |

*Note*. The pictures are ordered from highest to lowest emotional reactivity.
