# Supplementary material for: Adaptation and psychometric evaluation of the Natural Disasters Picture System (NDPS) to the Spanish population
Source: Front Psychol. 2026 Sep 16;17:1911152. doi: 10.3389/fpsyg.2026.1911152 (PMC13624170; doi:10.3389/fpsyg.2026.1911152)
Supplement: Supplementary file 6 [file Supplementary_File_6.doc]

**Appendix 5**

**Figure 1.** Importance of Predictor Variable (Random Forest).

*Note*. Bar chart representing the Importance of Score of each dimension in the Random Forest model for emotional cluster prediction.

**Figure 2**. Estimated Marginal Means of Valence by cluster (MLM).

*Note*. Graph of Estimated Marginal Means (EMMs) for the dimension Valence predicted by the emotional cluster based on MLM. Graph generated by RStudio (37).

**Figure 3**. Estimated Marginal Means of Arousal by cluster (MLM).

*Note*. Graph of Estimated Marginal Means (EMMs) for the dimension Arousal predicted by the emotional cluster based on MLM.

**Figure 4**. Estimated Marginal Means of Dominance by cluster (MLM).

*Note*. Graph of Estimated Marginal Means (EMMs) for the dimension Dominance predicted by the emotional cluster based on MLM.

**Figure 5.** Estimated Marginal Means of Certainty by cluster (MLM).

*Note*. Graph of Estimated Marginal Means (EMMs) for the dimension Certainty predicted by the emotional cluster based on MLM.

**Figure 6**. t-SNE visualisation.

*Note*. Each point represents a picture, and its colour represents the original cluster from Merlhiot et al. (2018), allowing it corresponds in the Spanish sample to be visualised.
