# Supplementary material for: Adaptation and psychometric evaluation of the Natural Disasters Picture System (NDPS) to the Spanish population
Source: Front Psychol. 2026 Sep 16;17:1911152. doi: 10.3389/fpsyg.2026.1911152 (PMC13624170; doi:10.3389/fpsyg.2026.1911152)
Supplement: Supplementary file 7 [file Supplementary_File_7.doc]

**Appendix 7**

To comply with the requirement for our modified questionnaire being openly available, we add in this Appendix 7:

a) A copy of our questionnaire sheet for each picture from Merlhiot et al. (2018): Figure 21 at the end of this Appendix.

b) Two links to Pavlovia used for participants who agree to answer the questionnaire (they have also been added as two “Data Review URL” links in the Attach Files step for submitting the paper), which contain the pictures:

<https://run.pavlovia.org/dmendoza36/sidn_a>

<https://run.pavlovia.org/dmendoza36/sidn_b>

c) A link to the freely available picture images database of Merlhiot et al. (2018):

[http://ndps.lapsco.fr](http://ndps.lapsco.fr/)

The previous link requires a username and password. We used the link Dr Merlhiot provided to directly download the ZIP file containing the full set of pictures.

**Figure 1**. Spanish-labelled questionnaire sheet for each picture.
